# Communication by Mothers with Breast Cancer or Melanoma with Their Children

**DOI:** 10.3390/ijerph10083483

**Published:** 2013-08-08

**Authors:** Rikki Gaber, Sapna Desai, Maureen Smith, Steve Eilers, Hanz Blatt, Yanina Guevara, June K. Robinson

**Affiliations:** 1 Department of Dermatology, Northwestern University Feinberg School of Medicine, Chicago, IL 60611, USA; E-Mails: rikki-gaber@northwestern.edu (R.G.); sapna.desai@fsm.northwestern.edu (S.D.); steven.eilers@northwestern.edu (S.E.); h-blatt@northwestern.edu (H.B.); yanina.guevara@northwestern.edu (Y.G.); 2 Center for Genetic Counseling, Northwestern University Feinberg School of Medicine, Chicago, IL 60611, USA; E-Mail: m-smith6@northwestern.edu

**Keywords:** health communication, breast cancer, melanoma, family communication, cancer prevention, early detection behaviors

## Abstract

Communication of familial risk of breast cancer and melanoma has the potential to educate relatives about their risk, and may also motivate them to engage in prevention and early detection practices. With the Health Insurance Portability and Accountability Act (HIPAA) privacy laws, the patient often becomes the sole communicator of such risks to family members. This study surveys mothers diagnosed with either breast cancer or melanoma and their adult children about their family communication style, knowledge of increased risk, and early detection practices. In both cancer groups, most mothers alerted their children of the risk and need for early detection practices. Breast cancer mothers communicated risk and secondary prevention with early detection by breast self-examination and mammograms whereas the melanoma mothers communicated risk and primary prevention strategies like applying sunscreen and avoiding deliberate tanning. Open communication about health matters significantly increased the likelihood that children engaged in early detection and/or primary prevention behaviors. Examining the information conveyed to at-risk family members, and whether such information motivated them to engage in early detection/prevention behaviors, is key to guiding better cancer prevention communication between doctors and patients.

## 1. Introduction

Early detection of melanoma and breast cancer improves survival. For melanoma patients diagnosed early with Stage IA melanoma, the survival rate 10 years after diagnosis was estimated as higher than 95%; but this rate declines to less than 60% for later stage diagnoses (Stage IIB, C), demonstrating the importance of secondary prevention such as skin self-examination (SSE) and visual inspection by a dermatologist [1]. In fact, visual skin examination is so significant that the National Institutes of Health (NIH) advises first-degree family members of patients with melanoma to see a dermatologist for a full body skin exam [2]. For breast cancer patients, if the disease is detected in Stage 0, the 5-year survival rate is 93%, while if detected in the later stages (IIIB–IV), the survival rate is less than 49% [3]. Despite the controversial efficacy of breast self-examination (BSE), monthly BSE for women are recommended from the onset of puberty to 40 years old. Clinical breast exams every three years for women between 20–39 years of age are also recommended, as well as yearly mammograms for women 40 years of age and older [4]. 

Primary and secondary prevention practices are recommended for those at risk of developing the melanoma and breast cancer due to hereditary or behavioral reasons (*i.e.*, deliberate tanning). Secondary prevention practices include early detection steps such as breast or skin self-examination, mammograms, doctor visits, or genetic testing for BRCA1 and BRCA2 (breast) and CDNK2A (p16, Leiden mutation for melanoma) [5]. Primary prevention steps include behavior changes such as stopping estrogen therapy or wearing sun block. Fortunately, both of these potentially deadly cancers have high survival rates for patients who detect and treat the cancer in its earlier stages. Thus, engaging in prevention practices is crucial for people at risk of developing these cancers. 

Mothers, who serve as the nexus of communication of health information for the family, can enhance awareness of the risk of developing these cancers and provide social support for primary and secondary prevention behaviors [6,7,8,9]. While knowledge about patterns of inheritance of breast cancer is often part of family culture, melanoma is commonly perceived as due to excessive ultraviolet light exposure [10,11,12]; however, melanoma can develop because of a CDNK2A mutation. When the hereditary predisposition to develop cancer is recognized by family members, then the illness of the mother can generate awareness of the heightened risk of developing the cancer among the children and other relatives [13]. Since Health Insurance Portability and Accountability Act (HIPAA) regulations place concerns around patient privacy and confidentiality, medical professionals are constrained in their ability to directly discuss health information with the relatives of the patient, therefore, the patient may be the sole disseminator of health information to family members [14]. 

Both breast cancer and melanoma disproportionately affect females. Furthermore, among all of the forms of cancer these are the only ones that empower the patient by providing a means for early detection by regular self-examination (breast and skin), thus, improving survival of the woman who chooses to engage in health promotion. Mothers, who are affected by the diseases, may play a pivotal role in enabling primary and secondary prevention practices among their children. If the child perceives themselves to be susceptible to the cancer, believes that the cancer has potentially serious consequences, believes engaging in early detection would be beneficial, and believes they can perform early detection practices, then the child is more likely to engage in such practices. The emotional impact of witnessing the mother’s sudden diagnosis with a potentially fatal disease may contribute to perception of risk [15]. If a woman believes herself to be at high risk for developing breast cancer, she is more likely to get mammograms earlier and more often than those women who do not perceive personal vulnerability [13]. Another way to enhance perception of personal and familial risk of developing a disease is genetic testing. 

This study evaluates the communication between mothers who had either breast cancer or melanoma and their adult children to discover: if the mother believed having the cancer implied that her children have a heritable risk to develop the same type of cancer; the preventative measures the mothers suggested; and whether the adult children perceived the risk and followed the counsel of their mothers. 

## 2. Methods

### 2.1. Recruitment of Participants

During the summer of 2012, recruitment involved calling mothers from a list of eligible patients compiled by Enterprise Data Warehouse of Northwestern University Feinberg School of Medicine using the following eligibility criteria: female participants (mothers) between ages 38 and 75 who had a diagnosis of any stage of melanoma or breast cancer within the five years preceding the study. To be eligible, women also had to have at least one son and one daughter between ages 18 and 50. Criteria for exclusion were inability to speak and understand English, or did not have children. Adult children were eligible if they had a mother with a diagnosis of breast cancer or melanoma within the last five years and were between ages 18 and 50. Adult children were excluded if they were unable to speak and understand English. 

Mothers were called by telephone twice within a time period of two weeks. The mother voluntarily provided the contact information for her adult child or children. Attempts to contact one adult child were made three times at weekly intervals or until they were reached. If a mother was reached by telephone, reasons given for choosing not to participate were recorded as: no eligible children; no biological children; no English spoken; no melanoma/breast cancer diagnosis; privacy concerns; mother could not speak over the phone; mother not interested; mother thought child would not be interested. 

If the mother was interested in participating, the survey was completed by telephone. The adult child was subsequently contacted and could choose whether to participate or decline. If the adult child was interested in participating, then he or she completed the survey by telephone. For instances where the mother provided contact information for multiple children, the survey was given to the first interested child reached.

### 2.2. Data Collection and Measures

Sequential and independent 10 min telephone interviews consisting of 29 items were conducted with the mother, followed by separate 10-minute telephone interviews consisting of 21 items with the adult child (see Appendixes A and B). Verbal informed consent was obtained from the mother and the adult child, and each was aware that the other was being interviewed. The survey delivered to the mothers involved questions aimed at revealing the openness of familial communication about health concerns, the reasons the mother had for talking to the adult child about his or her increased risk for cancer, and whether they advised their child to engage in any early detection practices. The survey delivered to the adult children comprised of questions aimed at revealing their perceptions of their own risk, and, if the adult child engages in prevention/early detection behaviors, what type of early detections. Answers to the free response questions were categorized once 20 in each sample size were gathered. The verbal consent, surveys and protocol were approved by the Institutional Review Board at Northwestern University.

### 2.3. Data Analysis Methods

Comparisons between breast cancer and melanoma mothers and children of these mothers were performed for each demographic variable. (Table 1) (Fisher’s exact test, *p* < 0.05). Comparisons for each variable for the mother (Table 2) and each variable for the child were performed. (Table 3) (Fisher’s exact test, *p* < 0.05). The engagement in early detection practices of children of mothers with breast cancer who reported open sharing of health information, was compared with those of mothers, who reportedly did not openly share health information (Table 3) (Fisher’s exact test, *p* = 0.05). 

**Table 1:** Demographic Variables of Participants.

Variables		Breast Cancer		Melanoma	
		Mothers n = 50 ^#^ (%)	Children n = 50 ^#^ (%)	Mothers n = 50 ^#^ (%)	Children n = 50 ^#^ (%)
**Marital status **					
	- Single	0 (0)	21 (42)	2 (4)	24 (48)
	- Married	36 (72)	25 (50)	37 (74)	25 (50)
	- Divorced	10 (20)	1 (2)	4 (8)	1 (2)
	- Separated	1 (2)	2 (4)	0 (0)	0 (0)
	- Widowed	3 (6)	1 (2)	7 (14)	0 (0)
**Education **					
	- Did not attend high school	0 (0)	0 (0)	1 (2)	0 (0)
	- Some high school	1 (2)	0 (0)	1 (2)	0 (0)
	- High school graduate	7 (14)	10 (20)	5 (10)	6 (12)
	- Some post-high school education	15 (30)	4 (8)	8 (16)	3 (6)
	- College graduate	16 (32)	25 (50)	15 (30)	24 (48)
	- Graduate degree	11 (22)	11 (22)	20 (40)	17 (34)
**Income **					
	- <$10,000	1 (2)	4 (8)	4 (8)	3 (6)
	- $10,000–19,999	0 (0)	3 (6)	0 (0)	1 (2)
	- $20,000–34,999	4 (8)	1 (2)	0 (0)	4 (8)
	- $35,000–50,999	4 (8)	6 (12)	7 (14)	5 (10)
	- $51,000–100,000	10 (20)	16 (32)	11 (22)	13 (26)
	- >$100,000	12 (24)	13 (26)	18 (36)	16 (32)
	- Not specified	19 (38)	7 (14)	10 (20)	8 (16)
**Occupational status **					
	- Student	0 (0)	7 (14)	0 (0)	5 (10)
	- Work part-time	4 (8)	10 (20)	8 (16)	4 (8)
	- Work full-time	7 (14)	27 (54)	15 (30)	34 (68)
	- Unemployed	3 (6)	2 (4)	2 (4)	5 (10)
	- Retired	24 (48)	0 (0)	21 (42)	0 (0)
	- Disabled	0 (0)	2 (4)	0 (0)	0 (0)
	- Homemaker	12 (24)	2 (4)	4 (8)	2 (4)
**Racial/ethnic background **					
	- White (non-Hispanic)	41 (82)	41 (82)	48 (96)	46 (92)
	- Black/African American	6 (12)	8 (16)	2 (4)	2 (4)
	- Multiracial	3 (6)	1 (2)	0 (0)	2 (4)
	- Hispanic/Latino	3 (6)	4 (8)	2 (4)	2 (4)
**Have health insurance **		50 (100)	50 (100)	49 (98)	47 (94)

Notes: Number in parenthesis indicates proportion of sample size; ^#^ Sum may be greater than 50 as discussions occurred more than once.

**Table 2:** Mothers’ rationale and means of informing children of risk.

Variables for Mother	Breast Cancer Pairs n = 50 ^#^ (%)	Melanoma Pairs n =50^ #^ (%)	*p* value
**Believes child can develop same type of cancer **	37 (74)	43 (86)	0.21
**Told child at increased risk **	45 (90)	45 (90)	0.99
- told daughters	45 (90)	45 (90)	0.99
- told sons	7 (14)	45 (90)	<0.0001
**Did not tell child at increased risk **			
- did not tell daughters	5 (10)	4 (8)	0.99
- did not tell sons	38 (76)	7 (14)	<0.0001
Reason told child *			
- knowledge of child’s risk based on:			
○ family history	17 (34)	5 (10)	<0.0001
○ similar physically ( body type, skin type)	6 (12)	40 (80)	<0.0001
○ doctor recommendation	15 (30)	0 (0)	<0.0001
- open communication	7 (14)	15 (30)	0.09
- child’s behavior (hormones, deliberate tanning)	8 (16)	28 (56)	<0.0001
**Reason did not tell child**			
- avoid causing anxiety	1 (2)	1 (2)	0.99
- child too busy to talk	3 (6)	3 (6)	0.99
- did not get a good opportunity to talk	5 (10)	3 (6)	0.72
**Advice given**			
- primary prevention			
○ sun protection	0 (0)	30 (6)	<0.0001
○ genetic testing	31 (62)	5 (10)	<0.0001
○ general health and wellness (smoking, diet, exercise)	16 (32)	13 (26)	0.66
- secondary prevention			
○ BSE, SSE **	12 (24)	4 (8)	0.05
○ doctor examination	13 (26)	8 (16)	0.33
○ mammogram	19 (38)	0 (0)	<0.0001
**Period of discussion ^#^**			
- at diagnosis	49 (98)	50 (1)	0.77
- before treatment	3 (6)	3 (6)	0.99
- during treatment	6 (12)	7 (14)	0.99
- after treatment	18 (36)	15 (30)	0.73
**Frequency of discussion with children ^^^**			
- once	14 (28)	15 (30)	0.99
- 2–10 times	43 (86)	39 (78)	0.74
- over 10 times	24 (48)	19 (38)	0.54
- not in the last year	14 (28)	13 (26)	0.99
**Mother believes child took advice **	42 (84)	39 (78)	0.82

Notes: *p* value calculated by Fisher’s exact test unless otherwise noted. * Sum may be greater than 50 as Mother has more than one reason. *p* value calculated by conditional binomial distribution. ^#^ Sum may be greater than 50 as discussions occurred more than once. *p* value calculated by conditional binomial distribution. ^^^ Sum may be greater than 50 as Mother may report discussion with more than one child. *p* value calculated by conditional binomial distribution; Number in parenthesis indicates proportion of sample size. ** BSE = breast self-exam, SSE = skin self-exam.

**Table 3:** Prevention Measures by Child of Mother with Breast Cancer or Melanoma.

Variables for Child	Breast Cancer Pairn = 50 ^#^ (%)	Melanoma Pair n = 50 ^#^ (%)	*p* value
**Child believes could get same type of cancer as Mother**	42 (84)	42 (84)	0.99
**Child feels open communication**	33 (66)	39 (78)	
**Child remembers advice**	44 (88)	41 (82)	0.58
- primary prevention			
○ sun protection (sunscreen, stay out of sun, no tanning beds)	0 (0)	25 (50)	<0.0001
○ genetic testing	6 (12)	1 (2)	0.11
○ general health and wellness (smoking, diet, exercise)	10 (20)	5 (10)	0.26
- secondary prevention			
○ BSE or SSE **	8 (16)	1 (2)	0.03
○ doctor examination	13 (26)	17 (34)	0.51
○ mammogram	31(62)	0 (0)	<0.0001
Child acted on advice by early detection *			
- BSE or SSE	33 (66)	15 (30)	0.0005
- doctor examination	21 (42)	24 (48)	0.69
- mammogram	29 (58)	0 (0)	<0.0001
**Child intends to act on advice by early detection**	2 (4)	11 (22)	0.01

Notes: *p* value calculated by Fisher’s exact test unless otherwise noted. * Sum may be greater than 50 as child may have taken more than one preventive step occurred more than once. *p* value calculated by conditional binomial distribution. Number in parenthesis indicates proportion of sample size. ** BSE = breast self-exam, SSE = skin self-exam.

Similarly, sun protection of children of mothers with melanoma who reported open sharing of health information, were compared with the responses of mothers who reported their family was not open to sharing health information (Fisher’s exact test, *p* = 0.05).

## 3. Results

### 3.1. Participants

**Figure 1:**
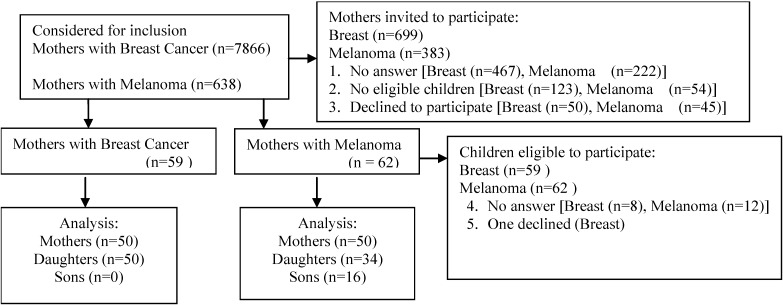
CONSORT diagram.

For this study, 50 melanoma mother-child pairs and 50 breast cancer mother-child pairs were surveyed. All eligible melanoma mother and child pairs reached by telephone participated in the study. Only daughters were interviewed in the breast cancer group (Figure 1). One of the contacted daughters in the breast cancer group failed to participate due to her being too busy. 

Mothers were between ages 45 and 75, and children were between ages 18 and 50. There were no significant differences in the age of the mothers and children between the two cancer groups. (Table 1) Both cancer groups were similar with respect to marital status, income, work status, race and ethnicity. Mothers with melanoma were significantly more educated than those with breast cancer (22% breast cancer mothers, graduate degree *vs.* 40% of melanoma mothers) (Fisher’s exact test, *p* < 0.05). Additionally, 99 out of the 100 mothers and 97 out of the 100 children surveyed had health insurance (Table 1). 

### 3.2. Mother’s Communication about Cancer Risk

Breast cancer mothers and melanoma mothers discussed the risk of cancer with their children (90% of mothers with either cancer) (Table 2). Mothers with breast cancer preferentially discussed the risk with daughters rather than sons (45 daughters and seven sons). Mothers with melanoma did not have a gender preference in the child with whom they discussed cancer risk (45 daughters and 45 sons) (Table 2). The primary reason for talking about cancer risk differed between those with melanoma and those with breast cancer (Table 2). Mothers with melanoma recognized that the child had the same type of skin as the mother (40/50) and that their child’s behavior placed them at risk (28/50). Mothers with breast cancer chose to discuss cancer risk with their children because of their personal knowledge of the child’s risk due to family history (17/50), and the doctor recommendation that the mother tell the adult child (15/30). Mothers with melanoma did not recall the doctor recommending that they discuss the possible risk of developing melanoma with the child (Table 2).

The mothers in the breast cancer group often encouraged their children to engage in early detection measures, such as self-examination or a clinical breast exam by a physician. They also mentioned the use of hormones to their daughters as a potential risk. The mothers in the melanoma group focused on primary prevention, such as sun protection, and cessation of deliberate tanning. Mothers from both groups made the effort to increase their children’s awareness of risk, and also of healthy behaviors to engage in. Discussions of risk of cancer between mother and adult child occurred most often at the time of diagnosis with second conversations occurring after treatment. 

### 3.3. Children’s Perceptions of Risk and Actions Taken

There was excellent agreement between the mother believing the child took the advice (42/50, 84% breast; 39/50, 78% melanoma) and the child remembering the advice (44/50, 88% breast, 41/50, 82% melanoma) (Cohen’s kappa = 0.97); however, the children did not act on the advice received from their mothers as often as the mothers believed. Less than half of the adult children in each cancer group acted on their mothers’ advice by seeing the doctor (21/50, 42% breast; 24/50, 48% melanoma) (Table 3). Whether the children acted on the mothers’ advice was associated with whether the mother and child both reported open communication about health matters. (Fisher’s exact test, *p* = 0.0001).

Children of breast cancer mothers breast cancer were more likely to take early detection measures (33/50 BSE, 21/50 doctor examination, 29/50 mammogram) than children of melanoma mothers (15/50 SSE; 25/50 doctor examination) (Table 3). Thirty-eight percent of the daughters started having a mammogram at a younger age than the general recommendation of age 40. Every adult child of a melanoma mother chose to do so at a younger age than the general recommendation of age 50 for having a skin examination. 

## 4. Discussion

When the mother communicated cancer risk and possible actions, the adult children took action based upon their perception of their personal risk and their knowledge of appropriate behaviors by engaging in primary and secondary prevention practices. While mothers with breast cancer and those with melanoma equally told their children of their increased risk, the reasons for informing the child differed. Knowledge of family history and early detection measures, including genetic testing, were communicated by breast cancer mothers to their daughters more than by melanoma mothers. Mothers that had breast cancer focused on early detection measures, such as BSE or mammograms, while mothers that had melanoma focused on primary prevention measures, such as wearing sun block or ceasing deliberate tanning. The emphasis on primary prevention advice by mothers with melanoma may be due to the perception that melanoma is caused by environmental factors, unprotected exposure to ultraviolet light. 

Children that had open conversations regarding cancer risk with their mothers took significantly more action to detect cancer than children who did not have open conversations with their mothers regarding cancer risk. Many of these children performed early detection behaviors sooner than the recommended ages for screening the general population. In other areas of health behavior, the child’s perception of parental behavior is the strongest predictor of the child’s behavior, thus, the perception of open communication by the parent about their cancer experience serves to inform and enable the child’s behavior. Communication between mothers and their children in this study was an effective way of providing information relating to cancer risk and the appropriate primary and secondary preventative measures. Family members may serve as a hidden workforce supporting medical decision making and adherence to ongoing preventative health care [16]. In this study, communication about breast cancer was greater between mothers and daughters than between mothers and sons. Breast cancer is associated with females and most research suggests that communication by females to males occurs less frequently than communication to a female relative [17]. Mothers pair bond with children, especially adult daughters, about health matters and invest considerable resources in the social relationship [18]. Eighty-eight percent of mothers diagnosed with breast cancer believed it was their personal responsibility to communicate cancer risks to their family [19]. It should not be assumed that at-risk family members are informed by relatives, some parents, particularly men, do not inform adult children [20].

Perceptions of familial risk are influenced by the physician informing mothers that the child may be at risk to develop the same type of cancer. While thirty percent of mothers with breast cancer recalled their doctor discussing the importance of the mother discussing it with their children, no mothers with melanoma recalled their doctor informing them of the potential for familial risk of melanoma. Communication of genetic risk, specifically discussing the option of genetic testing by the mothers in the breast cancer group may have activated adult children to perform early detection for breast cancer more often than was achieved by adult children of those with melanoma. This study identified the need for physicians providing care to mothers with melanoma to learn how to educate the mother about discussions with their children. Since first-degree relatives of melanoma patients with familial melanoma syndrome have a 10% incidence of developing melanoma, children of melanoma patients should be advised to perform SSE and to see a physician for a skin examination [21]. 

The limited sample of women patients and their adult children were of higher socio-economic status than the general population, which limits generalizability. Nearly all of the adult children had health insurance, making their access to healthcare and preventative screenings perhaps easier than for those without insurance. Access to healthcare for the children of the mother communicating the risk of cancer and providing knowledge about prevention and the children’s engagement in early detection practices is influenced by health insurance. 

## 5. Conclusions

Family communication by the mother with her children can provide valuable knowledge and enhance the personal relevance of cancer prevention for the children at risk. Open communication between mothers and their children concerning health matters may be enabled by the physician encouraging the patient to discuss her cancer experience with the family. The physician may also suggest visiting trusted websites for additional information or provide written materials on early detection practices, involving both self-examination and doctor examinations. The recent emphasis on person- and family-centered care as a major initiative of the National Priorities Partnership of the National Quality Forum suggests an appreciation of family relevance in health reform and quality improvement [22]. Physicians, who care for melanoma patients, need to be trained to engage mothers in discussing early detection practices with their children. Programs to train mothers in how to effectively communicate familial health risks could prove helpful in motivating the children to be proactive about cancer prevention. Physician- patient communication may be improved to ensure that patients are well-informed of the familial risk and to encourage them to communicate this risk to family members. Physicians, who care for mothers with melanoma, can help improve the communication competency of mothers with melanoma with their children.

## Appendix

### A. Mother Interview

(1) *How old are you? ___________ years old*
  - 35–44
  - 45–54
  - 55–64
  - 65–75
(2) *How many adult living children do you have? _______*_
(3) *How many boys?* ______
(4) *How many girls? ___*___
(5) *In general, how would you describe your family’s attitudes about discussing health matters?*
  - do not share health matters
  - share some information that is not too personal
  - share just about everything
  - discuss everything
(6) *Do you believe that is it possible that your children could develop the same type of cancer that you had? *
  - Yes
  - No
  - Don’t know
(7) *Have you told any of your children that they are now at greater risk of developing the same type of cancer that you had?*
  - Yes
  - No
(8) *Did your child advise you about getting tested?*
  - Yes
  - No
(9) *Please give me some reasons why you chose not to tell your children that they may develop the same type of cancer that you had.*
  - Did not want to upset them
  - Children are too busy to talk to
  - Family does not discuss health matters
  - Doctor should tell them
  - Did not get the opportunity
  - Child knows because child is in the healthcare field
If you told your adult children about the possibility of developing cancer, how many adult children did you tell?
(10) ____ *Daughters (write in a number)*
(11) _*____ Sons (write in a number)*
(12) *How did you choose which children to tell about the possibility of developing cancer?*
  - daughter/son lives near me
  - Children are too busy to talk to
  - have a good relationship with the child
  - daughter/son has children—desire to prevent grandchild from getting cancer e.g., stay out of sun, eat healthy, get genetic testing done
  - Told all children because of family history
  - Told all children because doctor recommended it
  - Told all children so that they take care of themselves
  - Just because
  - Gender preference-girl
  - Gender preference-boy
  - Potential genetic risk
(13) *Do your children engage in behaviors that you think make them more likely to develop cancer?*
  - No, no such behaviors
  - Daughter/son has an unhealthy diet
  - Daughter/son doesn’t exercise
  - Daughter/son goes to tanning salons
  - Daughter/son has been exposed to radiation
  - Daughter/son takes hormones (hormone replacement therapy, oral contraceptives, *etc*…)
  - Lot of sun exposure at any point in their lives
  - Stressful life
  - Smoker
(14) *What advice did you give them about the cancer?*
  - Go see doctor (detection)
  - Stay out of the sun/cover up (prevention)
  - Wear sunscreen (prevention)
  - Be aware of your skin/health (prevention)
  - Get tested/screened (*i.e.*, mammograms) (detection)
  - Self-exams (detection)
  - Don’t go to tanning salons (prevention)
  - General health and wellness (*i.e.*, no smoking, eat healthy, exercise, no hormones, positive attitude) (prevention)
  - Possible genetic risk (detection)
  - Consider mastectomy (detection)
(15) *Did you suggest skin or breast self-examination to your child/children?*
  - Yes
  - No
(16) *Did you suggest going to see their doctor?*
  - Yes
  - No
(17) *Did you suggest finding out more information? (i.e*., *on the web, library, print, books, magazines)*
  - Yes
  - No
(18) *Did you suggest the possibility of getting genetic testing?*
  - Yes
  - No
(19) *When during your care for cancer did you discuss early detection practices with your child?*
  - At time of diagnosis
  - Before treatment
  - During treatment
  - After treatment
(20) *Do you think your child took your advice?*
  - Yes
  - No
(21) *Did you talk to your child about what prevention steps they might take? (*e.g., *making doctor appointments, examining skin or breasts on a regular basis)*
  - Yes
  - No
(22) *Over the past year, about how many times have you discussed your children’s risk for cancer with them?*
  - Once
  - 2–10 times
  - Over ten times (nagged)
  - Not discussed in the past year
(23) *What is your marital status?*
  - Married
  - Never married
  - Divorced
  - Separated
  - Widowed
(24) *What is the highest level of education you have received?*
  - Did not attend high school
  - Some high school
  - High school graduate
  - Some post-high school education
  - College graduate
  - Graduate degree
(25) *What is your current occupational status?*
  - Student
  - Work part-time
  - Work full-time
  - Unemployed
  - Retired
  - Disabled
  - Homemaker
(26) *Which of the following categories best describes your annual household income? Stop me when I get to the one that best fits.*
  - Less than $10,000
  - $10,000 to $19,999
  - $20,000 to $34,999
  - $35,000 to $50,999
  - $51,000 to $100,000
  - Over $100,000
  - Prefer not to answer
(27) *Which of the following describes your racial background?*
  - Caucasian/White
  - Black or African American
  - Asian
  - Native Hawaiian or other Pacific Islander (please specify: ___________________)
  - American Indian or Alaska Native
  - Multiracial
  - Other (please specify: ____________________________________)
(28) *Do you consider yourself to be Hispanic or Latino?*
  - Yes
  - No
(29) *Do you have health insurance?*
  - Yes
  - No

### B. Adult Child Interview

(1) *How old are you? ________ years old*
  - 18–24
  - 25–29
  - 30
  - 31–39
  - 40
  - 41–50
(2) *Participant is:*
  - Daughter
  - Son
(3) *In general, how would you describe your family’s attitudes about discussing health matters?*
  - do not share health matters
  - share some information that is not too personal
  - share just about everything (=pretty open)
  - discuss everything
(4) *Do you remember your Mother telling you that you could develop the same type of cancer that she had?*
  a. Yes
  b. No
  If no—go to question 10 and continue from there
(5) *What advice did your Mother give you about the cancer?*
  - Go see doctor (detection)
  - Stay out of the sun (prevention)
  - Wear sunscreen (prevention)
  - Be aware of your skin and health (prevention)
  - Get tested (*i.e.*, mammograms) (detection)
  - Don’t go to tanning salons (prevention)
  - Possible genetic risk (detection)
  - Self-exams (detection)
  - General health and wellness (prevention)
  - Consider mastectomy (detection)
(6) *Did your Mother suggest that you perform skin or breast self-examination?*
  - Yes
  - No
(7) *Did your Mother suggest going to see your doctor to learn about the type of cancer that she had?*
  - Yes
  - No
(8) *Did you talk to a family member that is a healthcare professional to learn more about the type of cancer she had?*
  - Yes
  - No
(9) *Did your Mother suggest finding out more information? (i.e.*, *on the web, library, print, books, magazines)*
  - Yes
  - No
(10) *Did your Mother suggest the possibility of getting genetic testing?*
  - Yes
  - No
(11) *Do you believe that is it possible that you could develop the same type of cancer that your Mother had?*
  - Yes
  - No
  - Don’t know
(12) *Have you taken steps to check for cancer early?*
  - Yes
  - No
  - Not yet, but intend to
  - (If no, go to question 18)
(13) *What steps have you taken?*
  - Breast self-examination
  - Skin self-examination
  - Doctor visit
  - Mammogram
  - Genetic testing
(14) *Have you continued with the cancer checks?*
  - Yes
  - No
  - Intend to
(15) *What is your marital status?*
  - Married
  - Never married
  - Divorced
  - Separated
  - Widowed
(16) *What is the highest level of education you have received?*
  - Did not attend high school
  - Some high school
  - High school graduate
  - Some post-high school education
  - College graduate
  - Graduate degree
(17) *What is your current occupational status?*
  - Student
  - Work part-time
  - Work full-time
  - Unemployed
  - Retired
  - Disabled
  - Homemaker
(18) *Which of the following categories best describes your annual household income? Stop me when I get to the one that best fits.*
  - Less than $10,000
  - $10,000 to $19,999
  - $20,000 to $34,999
  - $35,000 to $50,999
  - $51,000 to $100,000
  - Over $100,000
  - Prefer not to answer
(19) *Which of the following describes your racial background? Stop me when I get to the one that best fits.*
  - Caucasian/White
  - Black or African American
  - Asian
  - Native Hawaiian or other Pacific Islander (please specify: __________________)
  - American Indian or Alaska Native
  - Multiracial
  - Other (please specify: ____________________________________)
(20) *Do you consider yourself to be Hispanic or Latino?*
  - Yes
  - No
(21) *Do you have health insurance?*
  - Yes
  - No
